# Analysis of Field Trial Results for Excavation-Activities Monitoring with φ-OTDR

**DOI:** 10.3390/s24186081

**Published:** 2024-09-20

**Authors:** Hailiang Zhang, Hui Dong, Dora Juan Juan Hu, Nhu Khue Vuong, Lianlian Jiang, Gen Liang Lim, Jun Hong Ng

**Affiliations:** 1 Institute for Infocomm Research (I^2^R), Agency for Science, Technology and Research (A*STAR), Singapore 138632, Singapore; zhang_hailiang@i2r.a-star.edu.sg (H.Z.); hdong@i2r.a-star.edu.sg (H.D.); vuong_nhu_khue@i2r.a-star.edu.sg (N.K.V.); jiang_lianlian@i2r.a-star.edu.sg (L.J.); lim_gen_liang@i2r.a-star.edu.sg (G.L.L.); 2 ST Engineering Urban Solutions Ltd., 6 Ang Mo Kio Electronics Park Road, Singapore 567711, Singapore; ng.junhong@stengg.com

**Keywords:** distributed fiber sensor, phase-sensitive optical time domain reflectometry, excavation monitoring, field trial, underground telecommunication fibers

## Abstract

Underground telecommunication cables are highly susceptible to damage from excavation activities. Preventing accidental damage to underground telecommunication cables is critical and necessary. In this study, we present field trial results of monitoring excavation activities near underground fiber cables using an intensity-based phase-sensitive optical time-domain reflectometer (φ-OTDR). The reasons for choosing intensity-based φ-OTDR for excavation monitoring are presented and analyzed. The vibration signals generated by four typical individual excavation events, i.e., cutting, hammering, digging, and tamping at five different field trial sites, as well as five different mixed events in the fifth field trial site were investigated. The findings indicate that various types of events can generate vibration signals with different features. Typically, fundamental peak frequencies of cutting, hammering and tamping events ranged from 30 to 40 Hz, 11 to 15 Hz, and 30 to 40 Hz, respectively. Digging events, on the other hand, presented a broadband frequency spectrum without a distinct peak frequency. Moreover, due to differences in environmental conditions, even identical excavation events conducted with the same machine may also generate vibration signals with different characteristics. The diverse field trial results presented offer valuable insights for both research and the practical implementation of excavation monitoring techniques for underground cables.

## 1. Introduction

With the rapid development and widespread popularity of the Internet, there has been a widespread adoption of underground deployment for telecommunication fiber cables within urban landscapes. Underground telecommunication cables can be easily damaged due to excavation activities. For example, there has been an increase in instances of telecommunication cable cuts in Singapore over the past few years. Between 2013 and 2018, there were a total of 23 incidents in Singapore [1,2]. Such incidents can result in large-scale disruptions of telecommunication, which will cause a variety of negative impacts on the end users as well as telecommunication operators [3], such as harming telecommunication operators’ brand reputation, affecting financial transactions, and the remote work practice. Thus, surveillance systems for early detecting excavation activities and avoiding large-scale disruption incidents are in demand in the market. The existing solutions such as patrolling or drone surveillance systems cannot achieve 24-h monitoring for all the routes of the fiber cables. Installing cameras or other sensors such as fiber Bragg gratings (FBGs) along all the routes of fiber cables will result in huge costs in scaling up the solution deployment. As a contrast, the solution based on phase-sensitive optical time-domain reflectometers (φ-OTDR) by using existing underground optical fibers as the sensing elements can be implemented quickly with a much lower cost [4,5].

The technique based on distributed acoustic sensing (DAS) utilizing standard optical fibers as distributed sensors is a promising solution to detect vibration signals at long distances along the standard fibers [6]. The most common DASs are based on φ-OTDR [7]. Other approaches such as those based on optical frequency domain reflectometry (OFDR) configurations [8,9], or time-gated digital optical frequency domain reflectometry (TGD-OFDR) were also employed to detect distributed vibrations [7]. However, OFDR configurations typically have a shorter sensing length and a lower detectable vibration frequency. TGD-OFDR can overcome the trade-off between spatial resolution and sensing length, but its setup is more complex and requires more sophisticated signal processing algorithms [10]. φ-OTDR based DAS is a promising long-range vibration monitoring technique that is particularly suited for applications requiring long-distance sensing and where cost and simplicity are priorities. It has been demonstrated for real-world applications such as highway vehicle detection [11], tide monitoring [12], earthquake detection [13], railway monitoring [14], pipeline surveillance [15], perimeter protection [16], and footstep detection for border security [17].

In the specific application of the detection and classification of excavation activities, numerous research efforts have been undertaken to combine artificial intelligence (AI) with φ-OTDR-based systems using underground telecommunication optical fibers to reduce nuisance alarm rates (NARs) in detection and to improve the accuracy in event classification [18]. However, variations in surrounding environmental conditions pose significant challenges for the widespread application of φ-OTDR technology for excavation monitoring in urban areas. The same type of excavation events such as road cutting or digging may exhibit different signal features or signatures when they are conducted at different locations or excavation sites. In addition, at the same location site, the signal signatures of the same types of excavation events are highly unlikely to be identical if they are carried out under different weather conditions (e.g., heavy rain versus sunny day) and/or different surrounding activities (e.g., a busy road junction when more traffic at peak hours or quiet traffic at midnight). Such complex environmental conditions have attributed significantly to the high NARs or lower recognition accuracy.

Therefore, there is a gap between the industry’s stipulated requirements and the reported event recognition accuracy. Successfully identifying various vibration events in a complex environment with a high recognition rate remains a challenging issue for future practical applications [19]. In addition, previously reported results of excavation monitoring are typically based on data collected from a single site study or single fiber cable [4,20,21,22,23,24,25]. For example, in [23], digging and hammering events were conducted at one quiet road with asphalt pavement. The hammering event showed 8.5 Hz fundamental frequency with many harmonic frequencies. The digging event showed broadband low-pass characteristics. In [24], DAS signals induced by excavator digging and hydraulic handheld hammering events over a buried fiber cable at urban and desert areas were analyzed. Only the time-frequency result of the signal collected at the desert area was analyzed, and the digging event showed broadband frequency response and strong mechanical at low frequences (<10 Hz). The handheld hammering events induced more energy at higher bandwidths in part due to its higher excitation rate. In [25], a digging event was conducted at a road with heavy activity at around 20 Hz. The DAS signal generated by excavation activities is strongly influenced by the surrounding environmental conditions. However, there are rarely reports comparing vibration signals of excavations which are located at different field sites.

In this paper, we present and analyze field trial results of monitoring various excavation activities using an in-house developed φ-OTDR interrogator and existing underground telecommunication cables at five different sites. The scheme of the φ-OTDR interrogator is based on intensity demodulation and direct detection. The rationale of selecting intensity demodulation-based φ-OTDR for monitoring excavation is presented in Section 2. All the data used in this paper were collected during actual excavation activities, rather than simulated events. To the best of our knowledge, this paper provides the greatest diversity in event types and field trial conditions for excavation monitoring with DAS. The field trial results and insights from this multiple-site study are dedicated to providing some reference for the research and practical implementation of excavation monitoring.

## 2. Sensing Principle, Rationale for Using Intensity-Based φ-OTDR, Experimental Setup, and Signal Processing Method

### 2.1. Sensing Principle

In φ-OTDR systems, an optical pulse of a highly coherent light pulse is launched into the fiber under test (FUT) and the Rayleigh backscattered (RB) light from a great number of different scattering centers within the pulse width interference coherently at the receiver. The phase and intensity of the RB light are sensitive to the local vibration. When external disturbances are applied to the FUT, the physical positions of the scatter centers in the affected region are slightly changed, which results in phase differences or intensity variations in the RB signal. Therefore, by demodulating the phase or intensity variations, the external vibrations can be monitored [26]. The demodulation methods of the φ-OTDR signal can be classified into two main groups, i.e., intensity demodulation and phase demodulation. For intensity demodulation, the vibration signal can be easily recovered, and the RB signal intensity has a nonlinear relationship with the vibration amplitude. Among the intensity demodulation-based schemes, φ-OTDR with direct detection has a simple structure and low cost, while phase-demodulation-based φ-OTDR normally requires coherent detection [27,28] or direct detection with an interferometer configuration [29,30].

### 2.2. Rationale for Using Intensity-Based φ-OTDR

In this paper, the φ-OTDR interrogator used is based on the scheme of intensity demodulation and direct detection. There are three reasons for choosing intensity demodulation-based φ-OTDR over phase demodulation-based φ-OTDR.

Firstly, in phase demodulation-based φ-OTDR, the coherent detection method strongly relies on the stability of the local oscillator (LO) arm and is associated with a higher noise [31]. Additionally, in direct detection with using an interferometer configuration, the instability of the delay arm can degrade φ-OTDR traces. In real-word applications, φ-OTDR interrogators are usually installed in a server room with a large vibration noise, e.g., vibration caused by the fans of the server cabinet. Therefore, phase demodulation-based schemes will be more vulnerable to perturbations than intensity demodulation schemes.

Secondly, we aim to monitor local excavation activities close to the underground optical fiber cables. Normally, the fiber cables are buried underground along roads and experience vibrations due to heavy traffic along the road throughout the entire fiber link simultaneously. The phase of the local-vibration-induced signal will be perturbed by the neighboring vibrations. In a phase-based φ-OTDR approach, phase information is not truly a local parameter, as it is subject to the hypothesis that phase disturbances occur only in the fiber section between pulses at two timestamps or two positions along the fiber [6,32]. Underground fiber cables are subjected to continuous vibrations over extended lengths due to traffic-induced vibrations or simultaneous construction activities at various locations above the cables. In such cases, the hypothesis becomes invalid, and the phase information becomes convoluted and difficult to distinguish. On the other hand, in intensity-based φ-OTDR systems, the intensity of the RB light is a local parameter which is independent of the vibrations experienced by other fiber sections beyond the pulse coved section [33,34].

Finally, the excavation induced vibrations on the ground that need to traverse through various layers such as mixed soil, cement pipe racks, and cable armor, before reaching the sensing fiber. Consequently, the signal collected by DAS becomes a nonlinear mixture [20], which depends on the uncertain local environmental conditions such as road and cable conditions. Therefore, for monitoring excavation, the collected DAS signal is nonlinear to the vibration amplitude in both intensity and phase-based demodulation.

In summary, intensity-based φ-OTDR is simpler, and sufficient for excavation detection.

### 2.3. Experimental Setup

**Figure 1:**
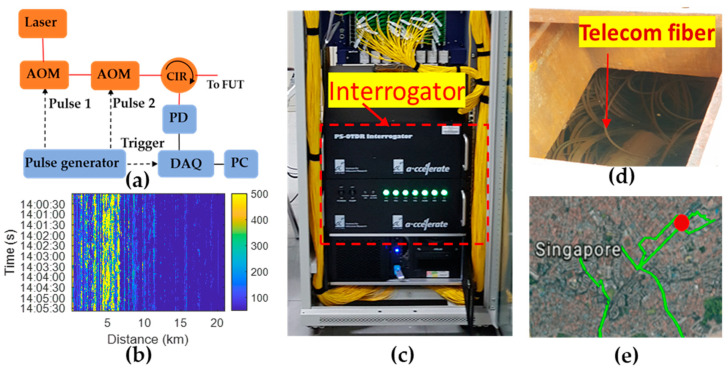
(**a**) Schematic diagram of the in-house developed φ-OTDR interrogator; (**b**) Example of a waterfall plot for 21 km fiber over about 5 min; (**c**) φ-OTDR interrogator was installed in a sever rack; (**d**) On-site photo of underground telecommunication fiber in the manhole; (**e**) Alarm of excavation event in GUI, the green curve represents the monitored fiber route. AOM: acousto-optic modulator, CIR: circulator, PD: photodetector, DAQ: data acquisition card, PC: computer.

The experimental setup for the field trials is shown in Figure 1. Figure 1a illustrates the schematic diagram of the in-house developed φ-OTDR interrogator. The laser source used was a RIO Grande high-power laser with low noise. It operated at a center wavelength of 1550.12 nm with a linewidth of less than 1 kHz and continuous-wave (CW) optical output of 29 dBm. The CW light was modulated into optical pulses with two acousto-optic modulators (AOMs), achieving a remarkably high total extinction ratio exceeding 100 dB. The rise/fall time of the first AOM (AA Opto-Electronics, Orsay, France, MT80-IIR30-Fio-PM5-J1-A-VSF) and second AOM (AA Opto-Electronics, Orsay, France, MT110-IIR25-Fio-PM5-J1-A-VSF) were 30 ns and 25 ns, respectively. The optical pulses were passed through the circulator (CIR) and launched into the FUT. The RB light from the FUT was detected using a photodetector (PD) (Keyang Photonics, Beijing, China, KY-PRM-10M-I-FC) featuring a 3 dB bandwidth of 10 MHz, and the resulting signal was captured by a data acquisition (DAQ) card (ADLINK, Singapore, PCle-9852). The control software of the φ-OTDR interrogator was developed using LabVIEW 2016. Figure 1b shows an example of a waterfall plot for monitoring 21 km of fiber over about 5 min. The front 7 km fiber was buried along an express road. The yellow colour represents strong vibrations caused by passing vehicles. In Figure 1c, an on-site photograph depicts the interrogator installed within a server rack in a server room. Figure 1d displays an on-site image of an underground telecommunication fiber situated within a manhole. Moving on to Figure 1e, the graphical user interface (GUI) of the system is presented, showcasing a monitored fiber route (depicted by a green curve) and a precisely identified excavation location.

### 2.4. Signal Processing Method

The data collected by the DAQ was shaped into a data matrix every second. Each column and each row of the data matrix represent the RB signal of a local position in time domain, and one RB trace along the fiber distance, respectively. The RB traces exhibit speckle-like patterns, indicating that the direct current (DC) components of the signals vary across different locations. Therefore, these DC components need to be filtered out. Additionally, to mitigate the influence of environmental noise, the low-frequency signal components in time domain need to be filtered out as well. If vibrations occur, peaks corresponding to the vibration locations will appear in the filtered RB trace. Typically, the root mean square (RMS) values of the filtered RB signal over a specific duration (e.g., one second) at different positions can be calculated to identify the vibration events. RMS values at the vibrating locations are markedly higher than those at quiet locations. The event location can be identified by localizing the position of the peak RMS value. In this paper, 60 s continuous signals at one position around the RMS peak for different events were compared and analyzed. Firstly, a 5 Hz high-pass digital filter was used to remove the DC and low frequency components of the time-domain signals. Then the frequencies of the 60 s signals are analyzed via Fast Fourier Transform (FFT). Furthermore, the time-frequency (TF) properties of the 60 s data are investigated through short-time Fourier transform (STFT), employing a short time window of 1 s and overlapping window of 0.5 s. The FFT results and STFT results are normalized to facilitate easier comparison.

## 3. Field Trials Details and Results

### 3.1. Field Trial Details

**Figure 2:**
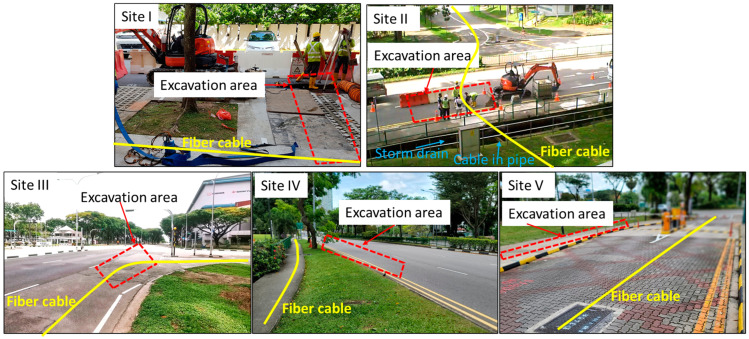
On-site photos of the five field trials for excavation detection. The yellow curves present the routes of underground fiber cables. Excavation activities were located in the areas marked by red dashed boxes.

Over the past few years, we have conducted numerous field trials to monitor excavation activities using the in-house developed φ-OTDR interrogator, which showed a stable performance and low system noise [35,36]. In this paper, we present the results of field trials conducted at five different sites, focusing on three primary types of excavation events: road cutting, hammering, and digging, as well as tamping operation. Five different mixed events (i.e., more than one primary event concurrently happened at the same location at the same time) at the fifth site are also investigated. The cutting, hammering and digging machines used in these five field trials are identical models. Field Trials IV and V employed the same set of machines. In addition, Field Trials IV and V utilized the same tamping rammer, which was different from that of field trial I and II. A camera was used to take on-site video for verifying events during all the field trials. The five field trial sites are labelled as Site I, Site II, Site III, Site IV, and Site V, respectively. Figure 2 shows the on-site photos of the five field sites. The excavation activities were located within the red dashed areas. The yellow curves represent the fiber routes of the underground fiber cables. Field trial I was conducted on 21 August 2020, and the excavation area was located at a walkway which was adjacent to a residential road. The surface of the excavation area was concrete pavement. Field trial II was conducted on 8 November 2021, and the excavation area was located at a main road. The surface of the excavation area was asphalt pavement. There was a storm drain alongside the road, and the fiber cable in a pipe ran across the storm drain. Field Trial III was conducted on 6 October 2022, and the fiber cable passed directly below the excavation area, which was located at a main road, and the surface of the excavation area was asphalt pavement. Field Trial IV was conducted on 9 March 2023, and the fiber cable was under the walkway alongside a main road, while the excavation area was located at the main road. Following Field Trial IV, Field trial V was conducted on 10 March 2023 by the same workers with the same machines. The excavation area was located on a walkway with concrete pavement. Table 1 lists the details of the five field trials as well as the settings of interrogator such as optical pulse width and the corresponding spatial resolution, pulse repetition rate, and spatial sampling resolution. It should be noted that in Field Trial III, the fiber cable was buried in soft soil directly at a depth of about 1m, while for the other four field trials, the cables were existing underground telecommunication fiber cables which were in pipes buried at a depth of about 1 to 1.5 m. For Field Trial III, the optical pulse width was set at 100 ns. In the other four field trials, due to the relatively larger total losses experienced in the fiber links, the optical pulse width was set to be 400 ns.

### 3.2. Field Trial Results

#### 3.2.1. Cutting Event

**Figure 3:**
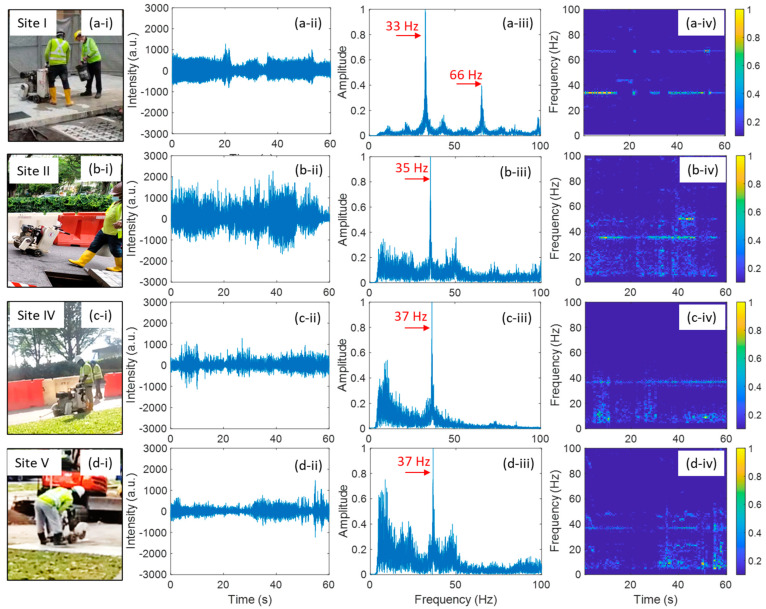
Field trial of cutting event: (**a**) at Site I, (**b**) at Site II, (**c**) at Site IV; (**d**) at Site V. (**i**), (**ii**), (**iii**) and (**iv**) show the cutting event scenes, cutting event signals in time domain, FFT results of the 60 s signals, time-frequency diagrams of the 60 s signal, respectively.

All except Field Trial III have cutting events. Figure 3 shows the field trial results of the cutting event for Field Trials I, II, IV, and V. The four field trials used cutting machines of the same model. Figure 3a–d illustrate the field trial results at Site I, Site II, Site IV, and Site V, respectively. Columns (i)–(iv) of Figure 3 correspond to the cutting event scenes, 60 s cutting event signals in time domain, FFT results of the 60 s signal, and TF diagrams of the 60 s signal, respectively. In the TF diagrams, each column is the FFT result of 1 s of temporal data. As can be seen from the FFT plots in column (iii) of Figure 3, the main vibration frequency of the cutting event is between 30 and 40 Hz. In Filed Trial I, the cutting event exhibited a frequency of about 33 Hz, along with a second-order harmonic frequency of about 66 Hz. In Field Trial II, IV, and V, there is no obvious harmonic frequency, and the vibration frequencies were about 35 Hz, 37 Hz, and 37 Hz, respectively. Comparing the TF diagrams in column (iv) of Figure 3, we can find that the signal of Field Trial I is obviously different from those of the other three field trials. When the cutting blade left the ground surface, i.e., the cutting action was paused, the vibration frequency peaks disappeared, e.g., around 18s in Field Trial I, as shown in Figure 3(a-iv). Normally, each of the cutting actions lasted more than 10 s.

The noticeable differences in frequency responses, whether involving harmonic frequencies or not, are likely due to the surface conditions of the cutting areas and the distances between the cable and the excavation site. In Field Trials I and V, the cutting took place on concrete pavement, while in Field Trials II and IV, it occurred on asphalt. The vibrations generated during the cutting events differ between asphalt and concrete pavements because of their distinct structural properties and material compositions. Concrete is typically stiffer and more rigid, whereas asphalt is more flexible. Consequently, vibration signals attenuate more quickly in asphalt than in concrete. Additionally, the distance between the cable and the excavation site in Field Trial V was larger than in Field Trial I, which is the primary reason why harmonic frequencies were not observed in Field Trial V, despite the road surface being concrete. This result highlights that varying environmental conditions can lead to different DAS signals for the same type of excavation events.

The cutting events in Field Trials IV and V exhibited very similar frequency responses, both featuring the same fundamental peak (FP) frequency of 37 Hz. This similarity is attributed to the use of the same cutting machine and the proximity of site IV to site V. In addition, the cutting event in Field Trial II exhibited a slightly different frequency response compared to Field Trials IV and V. One possible reason is that the cutting machine used in Field Trial II was not the exact same machine as in Field Trials IV and V.

#### 3.2.2. Hammering Event

**Figure 4:**
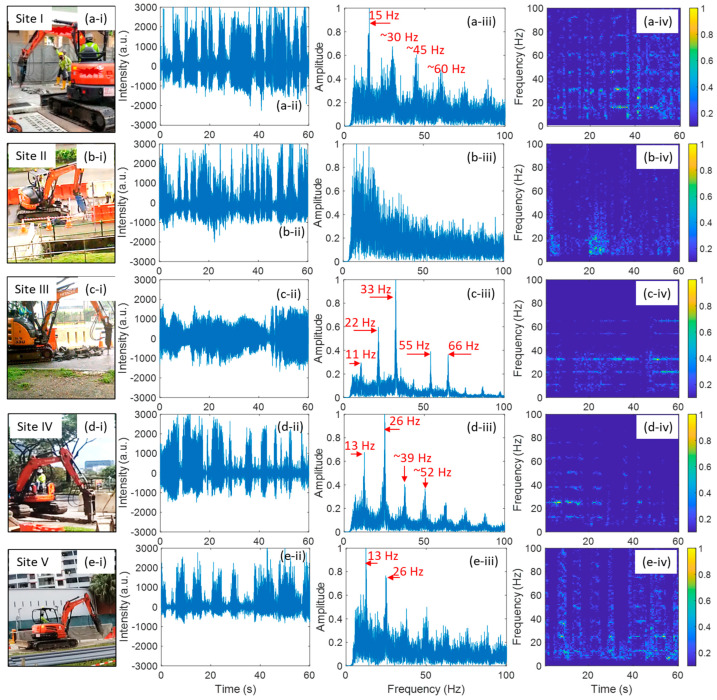
Field trial of hammering event: (**a**) at Site I, (**b**) at Site II, (**c**) at Site III, (**d**) at Site IV; (**e**) at Site V. (**i**), (**ii**), (**iii**) and (**iv**) show the hammering event scenes, hammering event signals in time domain, FFT results of the 60 s signal, time-frequency diagrams of the 60 s signal, respectively.

All the five field trials have hammering events, and their analysis is presented in Figure 4. Typically, hammering events generate much stronger vibrations compared to cutting events, leading to a broader range of harmonic frequencies in the DAS signal. The temporal signal of hammering events showed obvious intermittent patterns. The duration of each hammering action was a few seconds, as shown in column (ii) of Figure 4. The intermittent patterns can also be observed in TF diagrams, as shown in column (iv) of Figure 4, except Field Trial II. Field Trial II exhibits a broadband frequency response distinct from the other four trials that show only a few harmonic frequencies, as depicted in columns (iii) and (iv) of Figure 4. The primary possible reason is that the environmental conditions surrounding the fiber cable at Site II differs significantly from those at the other four sites. As shown in Figure 2, a storm drain was located alongside the excavation area at Site II, and the fiber cable in a pipe ran across the storm drain. In contrast, the fiber cables in the other four field trials were all buried underground. The spatial resolution of the DAS sensing signal for Field Trial II was about 40 m, which means that the collected signal also contained the RB light reflected from the fiber segment crossing the storm drain. The storm drain likely acted as a cavity, and the hammering event induced strong vibrations that could propagate and reflect along the cable segment crossing the storm drain. The frequency responses of the fiber cable segment crossing the storm drain were different from that of the buried underground segment. Consequently, the storm drain resulted in a very different frequency response with the other four Field Trials. Field Trial I, III, IV and V show similar yet different frequency patterns with a few harmonic frequencies. In Field Trial III, the fiber cable was buried directly in the soil, which exhibits the lowest fundamental frequency, i.e., about 11 Hz. Due to proximity of Site IV and Site V, the similar surrounding environmental conditions of the fiber cable led to very similar FFT results, as illustrated in Figure 4(d-iii,e-iii). Except for Field Trial II, the fundamental frequency of hammering events is between 10 and 15 Hz.

#### 3.2.3. Digging Event

**Figure 5:**
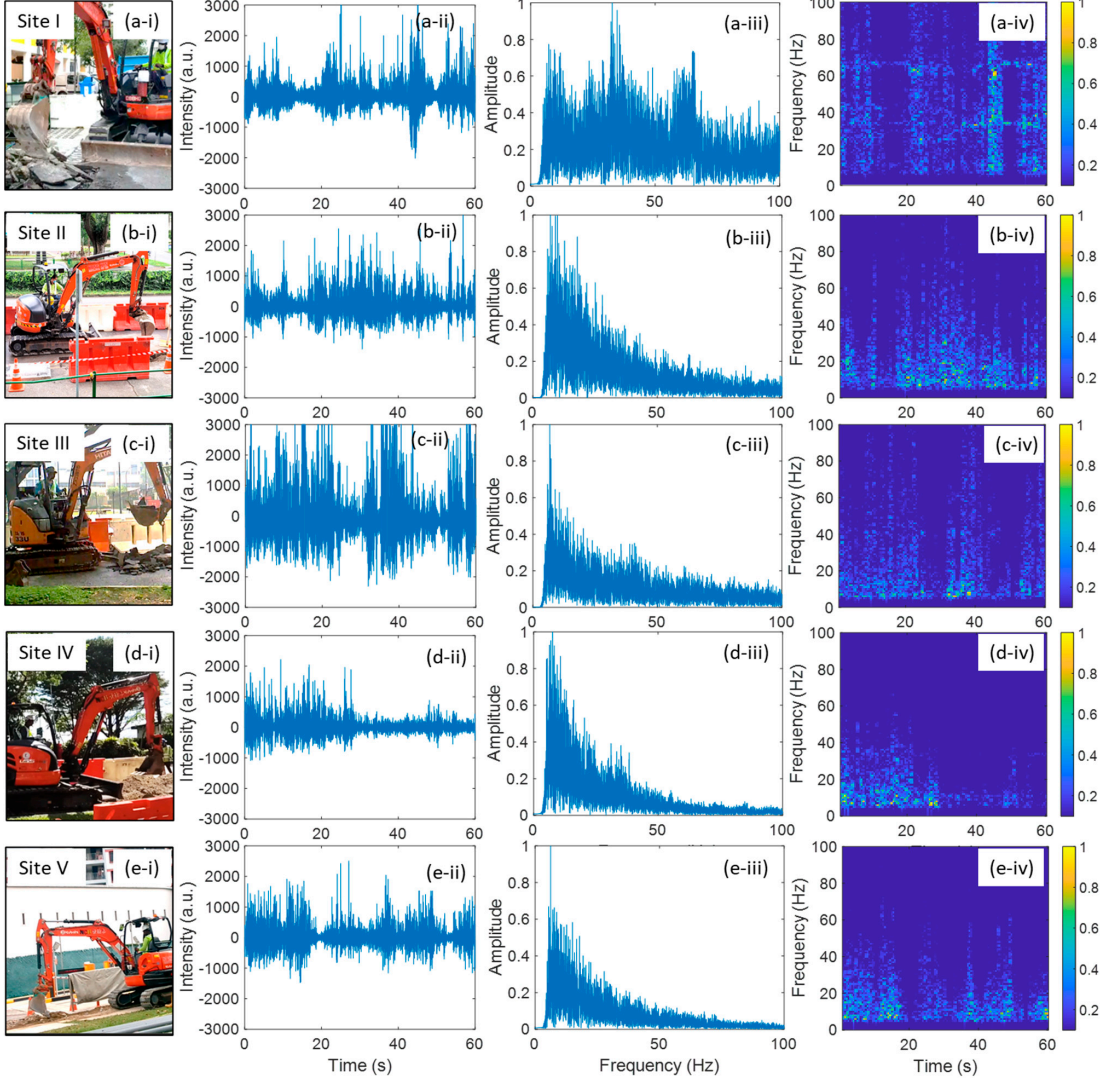
Field trials of digging event: (**a**) at Site I, (**b**) at Site II, (**c**) at Site III, (**d**) at Site IV; (**e**) at Site V. (**i**), (**ii**), (**iii**) and (**iv**) show the digging event scenes, digging event signals in time domain, FFT results of the 60 s signal, time-frequency diagrams of the 60 s signal, respectively.

Data collected while digging the road’s surface was chosen for analyzing digging events. The results of the digging events for all five field trials are depicted in Figure 5. In contrast to the cutting and hammering events, digging events did not exhibit distinct features in either FFT results or TF results. All the vibration signals generated by digging in the five field trials exhibit broadband spectrums, as depicted in column (iii) of Figure 5. Notably, Field Trial I showed a slight deviation from the remaining four trials, likely due to differing environmental conditions. The primary reason for the broadband frequency spectrum is that the digging process involved rapid changes in forces and motions, such as digging, scaping, lifting, and dumping. These dynamic processes contributed to a wide range of vibration frequencies.

#### 3.2.4. Tamping Event

**Figure 6:**
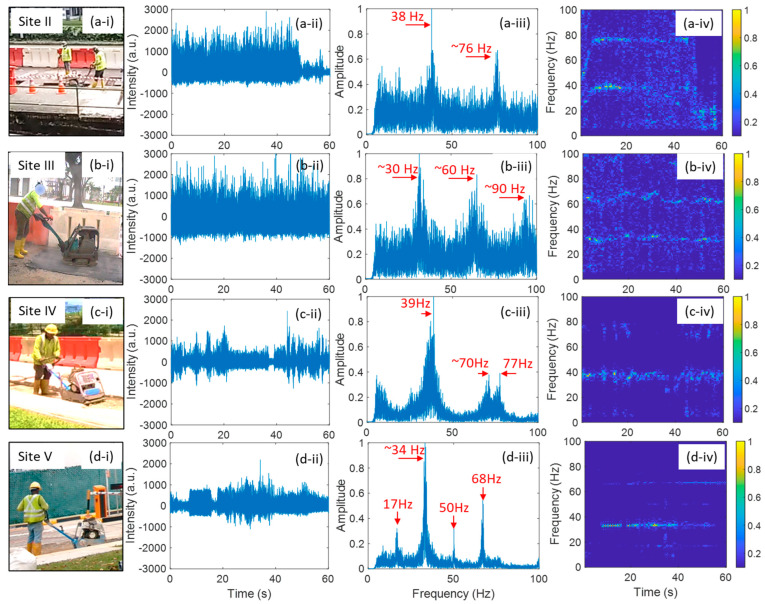
Field trial of tamping events: (**a**) at Site II, (**b**) at Site III, (**c**) at Site IV; (**d**) at Site V. (**i**), (**ii**), (**iii**) and (**iv**) show the tamping event scenes, tamping event signals in time domain, FFT results of the 60 s signal, time-frequency diagrams of the 60 s signal, respectively.

Figure 6 shows the field trial results of tamping events at Site II, III, IV and V. Typically, each continuous action of tamping lasted tens of seconds. The tamping events in all four field trials generated harmonic vibration frequencies, as shown in the FFT results depicted in column (iii) of Figure 6. The fundamental vibration frequencies of tamping events for Field Trials II, III and IV were between 30 and 40 Hz. In Field Trial V, the dominant frequency components were approximately 34 Hz and 68 Hz, with occasional appearances of a frequency component around 17 Hz, as depicted in Figure 6(d-iv). Despite the similar environmental conditions and the use of the same tamper in Field Trials IV and V, the frequency spectrums of the collected signals do differ, as shown in Figure 6(c-iii,d-iii). These differences highlight the importance of considering local site characteristics, such as soil composition, subsurface structure, and tamping mechanics when interpreting the DAS signal. Slight variations in the tamper operation, such as the force applied or the angle of impact, could also result in different vibration patterns. Even if the tamper machine is the same, differences in how the tamping action is carried out could affect the energy distribution and frequency content.

#### 3.2.5. Mixed Events

**Figure 7:**
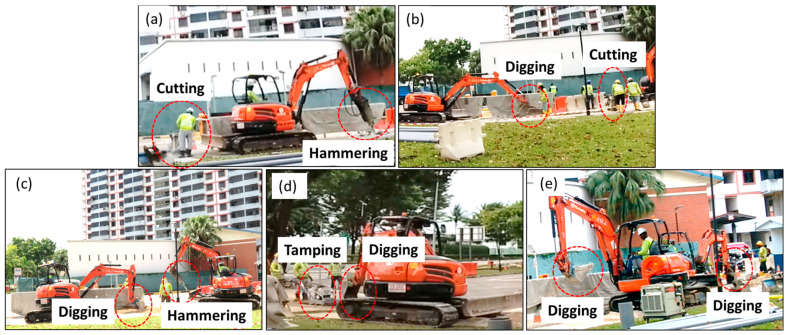
On-site photos of mixed events at Site V: (**a**) cutting and hammering, (**b**) cutting and digging, (**c**) hammering and digging, (**d**) digging and tamping, (**e**) digging and digging.

**Figure 8:**
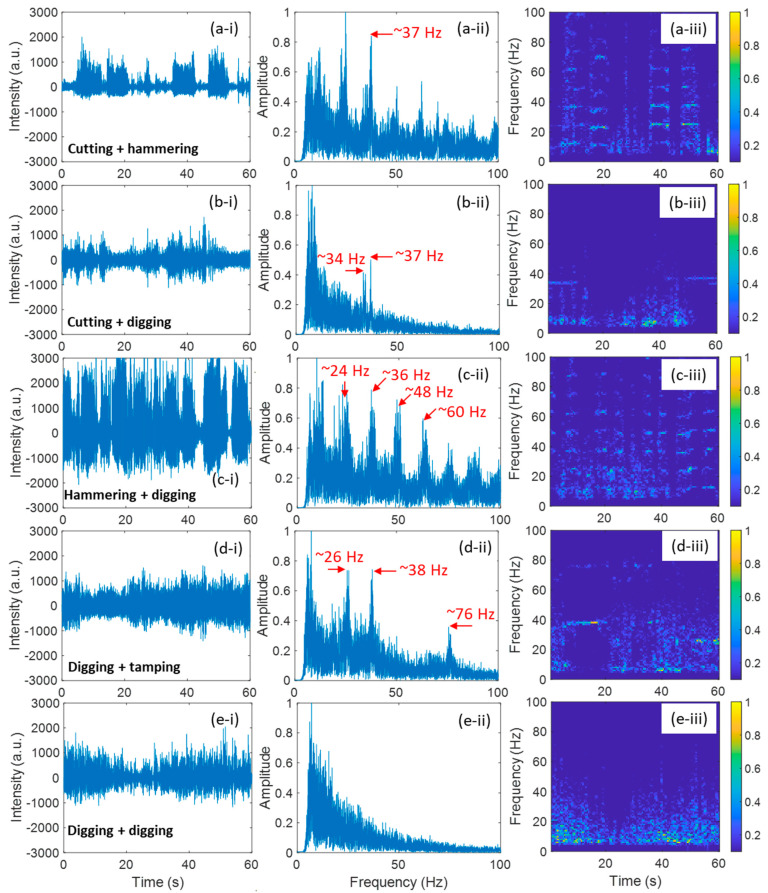
Results of mixed events at Site V: (**a**) cutting and hammering, (**b**) cutting and digging, (**c**) hammering and digging, (**d**) digging and tamping, (**e**) digging and digging. (**i**), (**ii**), and (**iii**) show the signal in time domain, FFT result of the 60 s signal, time-frequency diagrams of the 60 s signal, respectively.

During the excavation process, different events sometimes occur simultaneously. Figure 7 presents on-site photos of five different mixed events that occurred at Site V, including cutting with hammering, cutting with digging, hammering with digging, digging with tamping, and digging with digging. The signal of these five different mixed events at Site V are analyzed and shown in Figure 8. Columns (i)–(iii) of Figure 8 illustrate the 60 s temporal signals, FFT results of the 60 s signal, and TF diagrams of the 60 s signal, respectively. Figure 8a shows the mixed events of cutting and hammering. Compared to the individual cutting event (Figure 3d) and individual hammering event (Figure 4e), we can find that hammering dominates the signal features in the time domain as well as the frequency domain. The frequency component of 37 Hz should be induced by cutting events. This highlights the fact that while one activity may dominate the overall signal, other concurrent activities can still introduce specific frequency features. Figure 8b shows a mixed event of cutting and digging with an FFT spectrum more similar to that of the individual digging event, as shown in Figure 8(b-ii). This suggests that the digging process, which produced low-frequency signals, had a more significant impact on the signal spectrum compared to cutting. However, the appearance of frequency components at approximately 34 Hz and 37 Hz, attributed to cutting, indicates that even in the presence of dominant digging signals, cutting can still contribute specific high-frequency components. Figure 8c shows a mixed event of hammering and digging. As the vibration caused by hammering was much stronger than digging, the signal feature is dominated by the hammering event. The dominance of hammering makes it challenging to detect the contribution of digging in the signal. This suggests that for mixed events involving significantly different vibration intensities, the weaker event may be masked in the time and frequency domains unless more advanced signal decomposition techniques are employed. Figure 8d illustrates a mixed event involving digging and tamping. In Figure 8(d-ii), several peaks are visible in the FFT spectrum, which are similar yet distinct from those observed in the FFT result of an individual tamping event, as shown in Figure 6(d-iii). The FFT result retained the key features of tamping, but was slightly different due to the presence of digging. The group of low frequencies around 10 Hz was generated by a digging event. This suggests that while tamping dominates certain frequency ranges, the presence of digging introduces lower-frequency components, leading to a more complex spectrum. Figure 8e shows a mixed event of digging and digging, its FFT result is very similar to that of an individual digging event. The similarity in the signal suggests that simultaneous occurrences of the same type of activity result in additive effects that reinforce the signal without introducing new frequency components.

## 4. Discussion

The field trial results showed clear differences in frequencies across the trials, attributed to environmental conditions and differences in equipment or its operation. Table 2 compares the frequency features of different individual events across different field sites and previous related studies. Since few prior studies have reported on the frequency characteristics of mixed events, Table 2 focuses solely on individual events and does not include mixed event data. The diverse frequency characteristics observed across field sites and in comparison with previous studies emphasize the complexity of excavation monitoring. Environmental conditions, equipment variations, and activity types all play crucial roles in shaping the frequency signatures of excavation events.

The signal features of the four different individual and five mixed events are summarized in Table 3. Based on the field trial results, several observations can be made:

1. Cutting events exhibited the most consistent characteristics, with a distinct narrow frequency peak typically around 35 Hz. These actions usually lasted tens of seconds continuously.
2. Hammering events typically produced signals with a few harmonic frequencies, with the fundamental frequency ranging from 11 Hz to 15 Hz. However, due to complex environmental conditions, hammering events can also result in signals with a wideband spectrum, as observed in Field Trial II.
3. Digging events generated wideband spectrums without a clear frequency peak.
4. Tamping events produced signals with harmonic frequencies, although the frequency peaks are not as distinct as those in cutting events. The fundamental frequency typically ranges from 30 to 40 Hz.
5. The same type of events conducted by the identical machines but at different locations can result in varying frequency spectra, as observed from the analysis for tamping events in Field Trial IV and V.
6. Due to non-linear mixture, mixed events may result in more complicated signal features.

The fundamental frequencies of the detected signal primarily depend on the event types and machine models, with harmonic frequencies arising from the vibration intensity. The detected frequencies are closely related to the machines in use. However, the physical properties of the medium through which the vibrations propagate, such as environmental conditions, can also influence the frequency response [20]. In summary, the same type of events may exhibit similar yet varied signal features, which depend not only on the event types and surrounding environments but also on the machine models. In addition, during the excavation process, various mixed events frequently happen, and the non-linear mixed signal of the mixed events varies with time, which results in complicated signal features. Some algorithms for separating mixed vibration signals have been proposed and demonstrated [20,22], but the performances are still not good enough for practical excavation monitoring systems.

When excavation events take place at relatively quiet locations such as those sites in the field trials of this study, the acoustic signal features can be extracted. However, if the excavation is carried out at a noisy location such as alongside the road with heavy traffic, or close to a construction site, the features may be merged into the strong background noises. Traditional signal processing methods cannot distinguish excavation activities from strong background noise. Nevertheless, DAS remains an ideal scalable and cost-effective technical solution for monitoring underground assets during excavation activities. In scenarios of strong background noise, the deployment of an artificial intelligence (AI) recognition model becomes imperative for event recognition, aiming to enhance detectability and to minimize the NAR. Moreover, a substantial volume of raw data might be essential for training the AI model and enhancing its adaptability across diverse environmental conditions. The work of using AI methods to distinguish excavation events across different environments will be the next step for our further research.

## 5. Conclusions

This paper discussed field trial findings concerning the monitoring of various excavation activities using intensity-based φ-OTDR with underground optical fiber cables across five different sites. We presented the rationale behind selecting intensity-based φ-OTDR over phase-based φ-OTDR for excavation monitoring in urban areas. Additionally, we analyzed and compared the signals generated by various excavation activities, such as cutting, hammering, digging, tamping, and five mixed events. The data used in this paper were collected during real excavation activities, but not simulated events. To our knowledge, this paper offers the most diverse range of excavation event types and field trial conditions for monitoring excavations. It is noted that the characteristics of the vibration signal generated by excavation events are influenced by both environmental conditions and the machinery used for excavation. Typically, fundamental peak frequencies of cutting, hammering and tamping events ranged from 30 to 40 Hz, 11 to 15 Hz, and 30 to 40 Hz, respectively. Digging events, on the other hand, presented a broadband frequency spectrum without a distinct peak frequency. In addition, mixed excavation events can lead to a more complex spectrum. Therefore, traditional signal processing methods encounter difficulties in distinguishing excavation events across various environmental conditions and machinery types. Leveraging AI to improve the differentiation of excavation events in diverse environments will be a key research focus for excavation monitoring

## Figures and Tables

**Table 1:** Details of the field trials.

Field Trials	Information of Field Trials	Event Types	Interrogator Setting
Field Trial I	Date: 21 August 2020 Fiber length: ~23 km, project site location: ~8.5 km Site condition: walkway located alongside residential road with normal traffic Surface of excavation area: concrete pavement Fiber cable was in a pipe buried underground	Cutting Digging Hammering	Optical pulse width: 400 ns Spatial resolution: ~40 m Pulse repetition rate: 1 kHz Sampling resolution: 1 m
Field Trial II	Date: 8 November 2021 Fiber length: ~21 km, project site location: ~9.2 km Site condition: main road with many vehicles; storm drain next to the excavation area Surface of excavation area: asphalt pavement Fiber cable was in a pipe buried underground	Cutting Digging Hammering Tamping	Optical pulse width: 400 ns Spatial resolution: ~40 m Pulse repetition rate: 1 kHz Sampling resolution: 1 m
Field Trial III	Date: 6 October 2022 Fiber length: ~1.4 km, project site location: ~800 m Site condition: main road with many vehicles; close to industry factories Surface of excavation area: asphalt pavement Fiber cable was buried in soil directly	Digging Hammering Tamping	Optical pulse width: 100 ns Spatial resolution: ~10 m Pulse repetition rate: 2 kHz Sampling resolution: 1 m
Field Trial IV	Date: 9 March 2023 Fiber length: ~12.3 km, project site location: ~9.1 km Site condition: main road with many vehicles Surface of excavation area: asphalt pavement Fiber was in a pipe buried underground	Cutting Digging Hammering Tamping	Optical pulse width: 400 ns Spatial resolution: ~40 m Pulse repetition rate: 1 kHz Sampling resolution: 2 m
Field Trial V	Date: 10 March 2023 Fiber length: ~12.3 km, project site location: ~9.2 km Site condition: entrance of carpark Surface of excavation area: concrete pavement Fiber was in a pipe buried underground	Cutting Digging Hammering Tamping	Optical pulse width: 400 ns Spatial resolution: ~40 m Pulse repetition rate: 1 kHz Sampling resolution: 2 m

**Table 2:** Comparison of frequency features of individual events across different field sites and prior works.

Field Sites	Cutting	Hammering	Digging	Tamping
Site I	FP frequency: ~33 Hz Harmonic frequency: 66 Hz	FP frequency: ~15 Hz With a few harmonic frequencies with narrow bands	Broadband frequency spectrum with three main frequency groups at around 10 Hz, 30 Hz and 60 Hz.	nil
Site II	FP frequency: ~35 Hz	Broadband frequency spectrum	Broadband frequency spectrum with strongest energy at around 10 Hz, and 3 dB bandwidth less than 30 Hz	FP frequency: ~38 Hz With harmonic frequencies
Site III	nil	FP frequency: ~11 Hz With a few harmonic frequencies with narrow bands	Broadband frequency spectrum with strongest energy at around 10 Hz, and 3 dB bandwidth less than 30 Hz	FP frequency: ~30 Hz With harmonic frequencies
Site IV	FP frequency: ~37 Hz	FP frequency: ~13 Hz With a few harmonic frequencies with narrow bands	Broadband frequency spectrum with strongest energy at around 10 Hz, and 3 dB bandwidth less than 30 Hz	FP frequency: ~39 Hz With harmonic frequencies
Site V	FP frequency: ~37 Hz	FP frequency: ~13 Hz With a few harmonic frequencies with narrow bands	Broadband frequency spectrum with strongest energy at around 10 Hz, and 3 dB bandwidth less than 30 Hz	FP frequency: ~34 Hz With harmonic frequencies
In desert (in 2024) [24]	nil	Note: handheld hammering Induced energy in the upper bandwidth part (typically >10 Hz)	Broadband frequency spectrum, with strong energy at low frequencies (<10 Hz)	nil
At road with asphalt pavement (in 2021) [23]	nil	FP frequency: ~8.5 Hz With a few harmonic frequencies	Broadband frequency spectrum with 3 dB bandwidth less than 100 Hz	nil
At road with asphalt pavement (in 2021) [25]	nil	nil	Broadband frequency spectrum with strongest energy at around 20 Hz	nil

**Table 3:** Signal features of different excavation events.

Event Types	Signal Features
Cutting	Fundamental frequency has a narrow band. FP frequency is between 30 and 40 Hz, e.g., 33 Hz, 35 Hz, 37 Hz May have harmonic frequency. Time domain: each action lasts continuously for tens of seconds
Hammering	Normally, frequency components consist of a few harmonic frequencies with narrow bands. FP frequency is between 11 to 15 Hz, e.g., 11 Hz, 13 Hz, 15 Hz However, the signal may have a wide frequency spectrum, e.g., in Field Trial II. Time domain: obvious intermittent patterns, each action lasts continuously for a few seconds.
Digging	Wide frequency spectrum Time domain: intermittent patterns, each action lasts a few seconds, normally with a shorter duration than hammering
Tamping	Typically, fundamental frequency covers a wider band than cutting and hammering events. FP frequency typically falls within the 30 to 40 Hz range, e.g., 30 Hz, 38Hz, 39 Hz. Time domain: each action lasts continuously for tens of seconds.
Cutting + Hammering	Hammering event dominates the signal features in FFT result and TF result
Cutting + Digging	Obviously different with cutting or digging in FFT result and TF result. Wide frequency spectrum with a few frequency peaks caused by cutting
Hammering + Digging	Hammering event dominates the signal features in FFT result and TF result
Digging + Tamping	Obviously different with digging or tamping in FFT result and TF result. A few frequency peaks caused by tamping can be observed
Digging + Digging	Very similar to individual digging event, i.e., wide frequency spectrum

## Data Availability

Data are unavailable due to the data policy of our institute but are available from the corresponding author on reasonable request.
